# Dataset of spark plasma sintering of Al—Zn—Sn alloy for soft solder application

**DOI:** 10.1016/j.dib.2019.103948

**Published:** 2019-04-23

**Authors:** O.P. Oladijo, A.P.I. Popoola, C.O. Ujah, M. Namoshe

**Affiliations:** a Department of Chemical, Materials and Metallurgical Engineering, Botswana International University of Science and Technology, Palapye, Botswana; b Mechanical Engineering Science Department, University of Johannesburg, Auckland Park Kingsway Campus, Johannesburg, South Africa; c Department of Chemical, Metallurgical and Materials Engineering, Tshwane University of Technology, Pretoria, South Africa; d Department of Mechanical, Energy and Industrial Engineering, Botswana International University of Science and Technology, Palapye, Botswana

**Keywords:** Spark plasma sintering, Soft solder, Micro hardness, Aluminium, Tin, Zinc

## Abstract

The conventional soft solder used in soldering electronic joints is made up of 63% Sn and 37% Pb. It has been established that Pb is harmful to the body. Therefore, there is an on-going research to find a replacement for Pb. Al—Zn—Sn alloy is considered here as a possible replacement for soft solder of Sn and Pb. Three compositions of the alloy (Al—7Zn—3Sn, Al—10Zn—5Sn, and Al—13Zn—7Sn) were sintered at the sintering temperatures of 300 °C and 350 °C (and the constant pressure of 40 MPa, time of 5 mins, a heating rate of 20 °C/mins). Temperature/displacement/time variation data during the sintering were collected. The three alloys were compared after sintering by checking their microstructure, their densities, their hardness, their porosity, and their tensile strengths. Results showed that Al—13Zn—7Sn sintered at 350 °C, 40 MPa, 5 mins, 20 °C/mins had the highest densification of 99.7%, the lowest porosity of 0.3%, least hardness and strength of 450.34 MPa and 147.84 MPa respectively.

**Table:** 

Subject area	Material science
More specific subject area	Powder metallurgy
Type of data	Table, graph
How data was acquired	The first six data (Table 1, Table 2, Table 3, Table 4, Table 5, Table 6) were generated by spark plasma sintering machine (KCE-FCT-HHPD 25, Germany), during the SPS of the Al—Zn—Sn composite. The density data was got using Archimedes principle. The Relative density was got by comparing the theoretical and actual densities. Porosity [%] = 100% - Relative density (%) [1] The hardness of the composite samples was conducted on Vicker's Micro hardness tester (FM-800, Japan) with 100 g force for the duration of 15s and spacing of 0.1 with 5 indentations. Tensile strength (Ts) [MPa] = (H/2.9) (n/0.217)^n^[2] Where H is hardness in [MPa], n is strain hardening coefficients of the material, (n = 0.16). H [MPa] = H [HV] x 9.807 [3] Where H [MPa] is the hardness in Mega Pascal, H [HV] is the Vicker's hardness
Data format	Raw, Computed
Experimental factors	Sintering temperature and concentration of Zn and Sn in the alloy were the two variable factors. Pressure, dwell time, and heating rate were constant factors. The sintering machine was initialized with the following parameters: vacuum pressure was 0.605 mbar (46.09%), the relative pressure was −500 mbar, and absolute pressure was 1.2 mbar.
Experimental features	The sintering was performed at room temperature of 27 °C; There was a total of six runs. Each run has its peculiar features based on the composition and sintering parameters (described in the experimental design and method). The sintered samples were immediately sandblasted after sintering to avoid graphite contamination. Graphite paper was used to shield the sample from the die.
Data source location	Department of Chemical, Metallurgical and Materials Engineering, Tshwane University of Technology, Pretoria, South Africa (Nano laboratory)
Data accessibility	All data are included in the article.
Related research article	C.O. Ujah, A. P. I. Popoola, O. M. Popoola, V. S. Aigbodion, Optimisation of spark plasma sintering parameters of Al-CNTs-Nb nano-composite using Taguchi Design of Experiment, The International Journal of Advanced Manufacturing Technology, 2018, https://doi.org/10.1007/s00170-018-2705-3[1]

**Table:** 

**Value of the data** • The data could be used to determine the response of the alloy being consolidated to the increase in temperature over time. • It could be used to study the displacement (change in shape) of the samples over time when heat is applied. • The data would help in predicting the best sample suitable to replace Pb solder based on their response to an increase in temperature and shape. • The hardness/porosity data would help to determine the samples that are soft for replacing Pb solder yet having low porosity (absence of voids and impurities).

## 1 Data

**Fig. 1:**
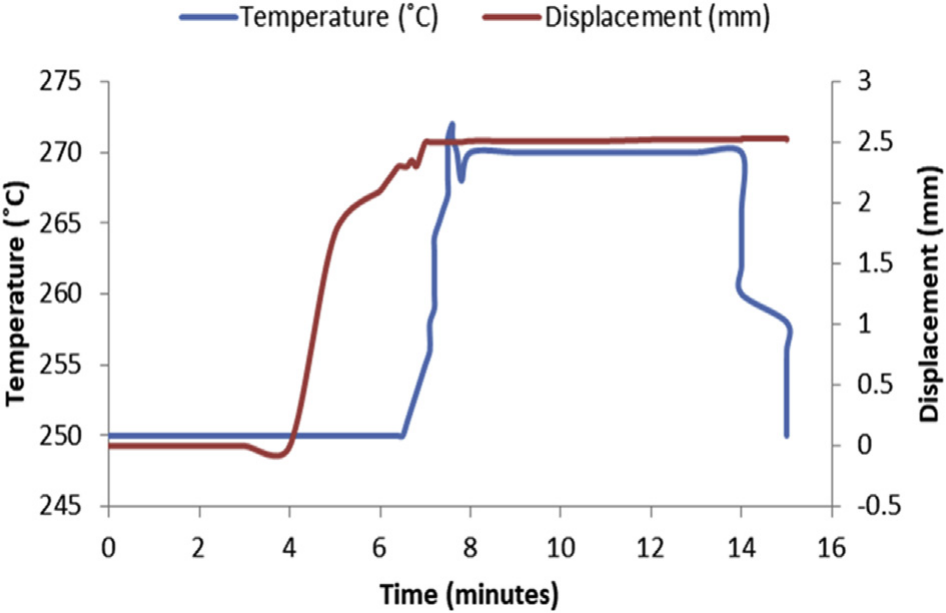
Temperature/displacement versus time profile for Al—7Zn—3Sn during SPS at 270 °C, 30 MPa, 5 mins and 20 °C/mins.

**Fig. 2:**
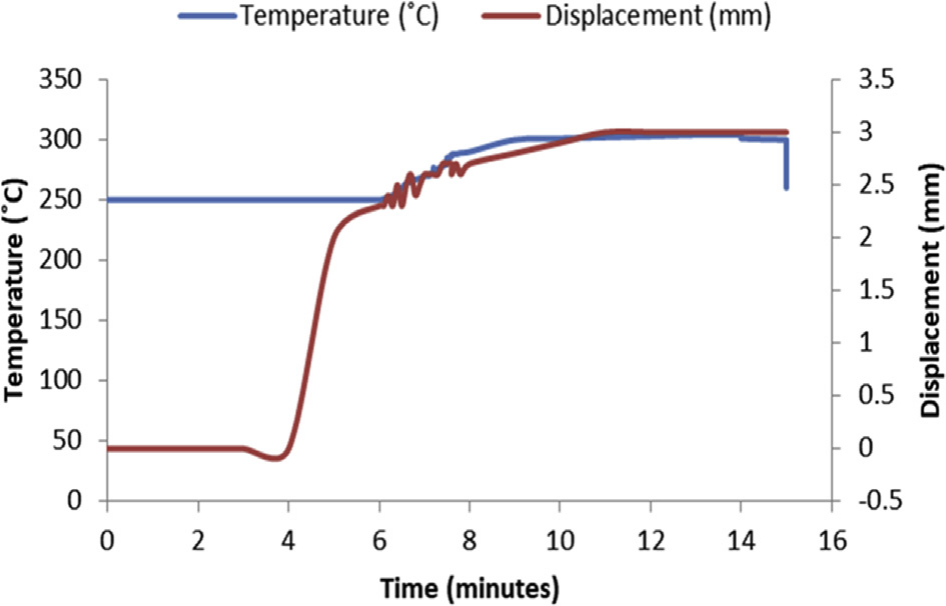
Temperature/displacement versus time profile for SPS of Al—7Zn—3Sn at 300 °C, 40 MPa, 5 mins and 20 °C/mins.

**Fig. 3:**
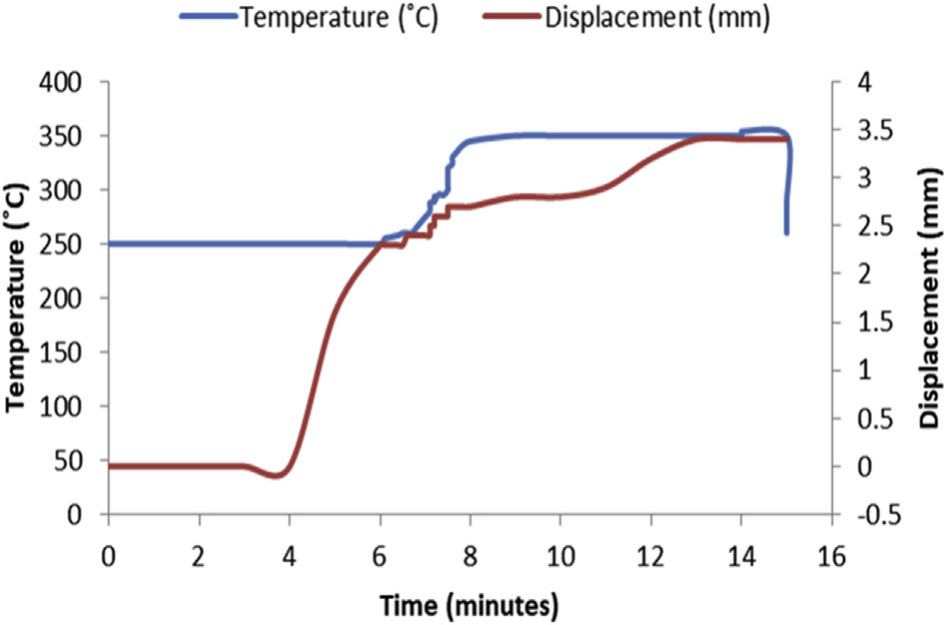
Temperature/displacement versus time profile for SPS of Al—7Zn—3Sn at 350 °C, 40 MPa, 5 mins and 20 °C/mins.

**Fig. 4:**
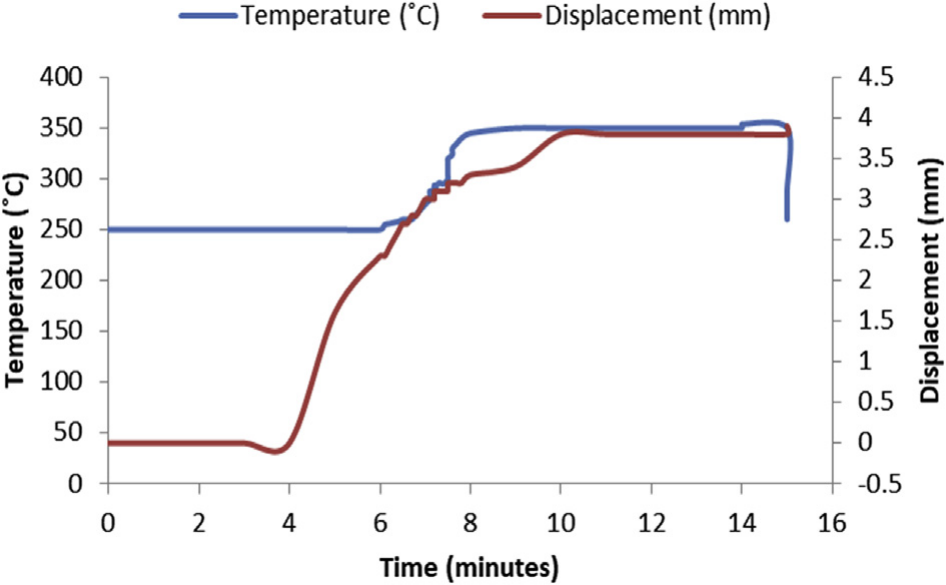
Temperature/displacement versus time profile for SPS of Al—10Zn—5Sn at 350 °C, 40 MPa, 5 mins and 20 °C/mins.

**Fig. 5:**
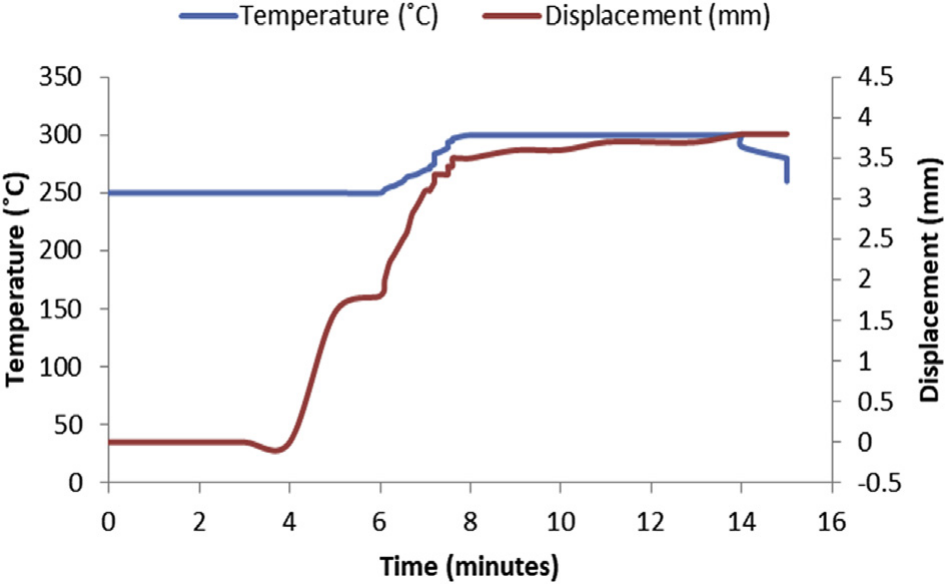
Temperature/displacement versus time profile for SPS of Al—13Zn—7Sn at 300 °C, 40 MPa, 5 mins and 20 °C/mins.

**Fig. 6:**
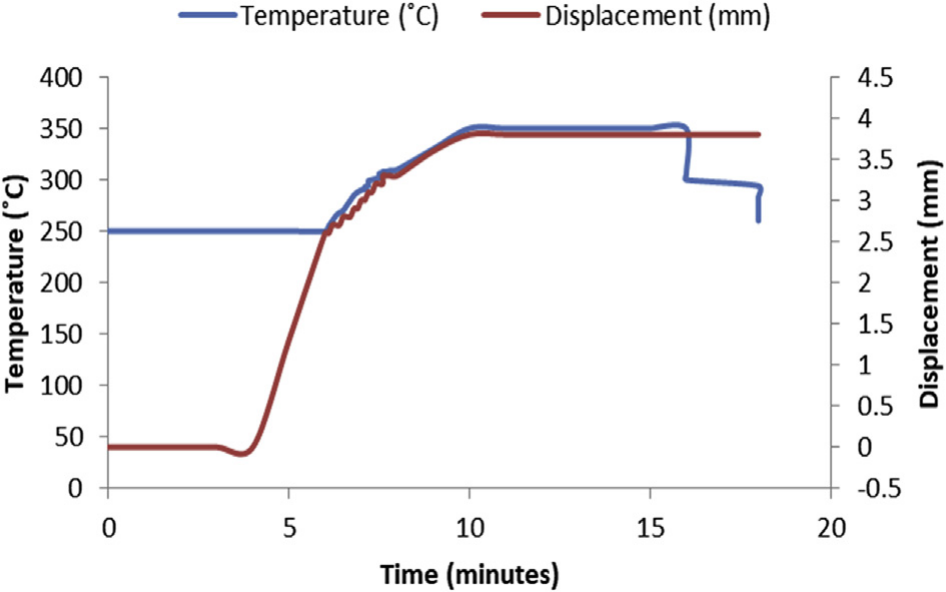
Temperature/displacement versus time profile for SPS of Al—13Zn—7Sn at 350 °C, 40 MPa, 5 mins and 20 °C/mins.

**Fig. 7:**
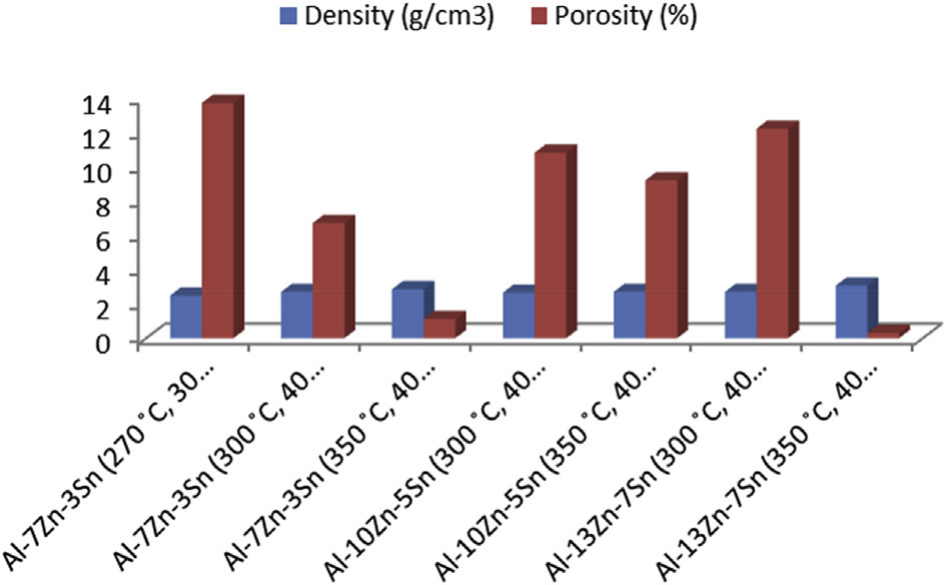
Plot of density/porosity profile for sintered samples.

**Fig. 8:**
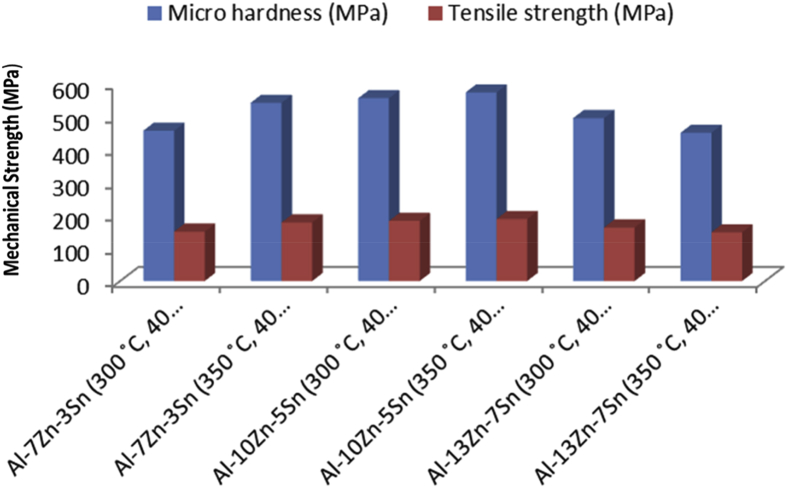
Plot of micro hardness and tensile strength of sintered samples.

The data in Table 1, Table 2, Table 3, Table 4, Table 5, Table 6 are the sintering temperature, sintering time and displacement of the samples during sintering. It displaced the overall factors from the beginning of the sintering to the finish. Fig. 1, Fig. 2, Fig. 3, Fig. 4, Fig. 5, Fig. 6 display the plot of the factors in Table 1, Table 2, Table 3, Table 4, Table 5, Table 6. Table 7 shows Density, Relative density and Porosity data of sintered samples (see Fig. 7). Table 8 is the computed Micro hardness and Tensile strength data (see Fig. 8).

**Table 1:** Temperature/displacement/time data for SPS of Al—7Zn—3Sn at 270 °C, 30 MPa.

Time (mins)	Temperature (˚C)	Displacement (mm)
0	250	0
1	250	0
2	250	0
3	250	0
4	250	0
5	250	1.75
6	250	2.1
6.1	250	2.15
6.2	250	2.2
6.3	250	2.25
6.4	250	2.3
6.5	250	2.3
6.6	251	2.3
6.7	252	2.35
6.8	253	2.3
6.9	254	2.4
7	255	2.5
7.1	256	2.5
7.1	257	2.5
7.1	258	2.5
7.2	259	2.5
7.2	260	2.5
7.2	261	2.5
7.2	262	2.5
7.2	263	2.5
7.2	264	2.5
7.3	265	2.5
7.4	266	2.5
7.5	267	2.5
7.5	268	2.5
7.5	269	2.5
7.5	270	2.5
7.5	271	2.5
7.6	272	2.5
7.6	271	2.5
7.7	270	2.5
7.8	268	2.5
8	270	2.51
9	270	2.51
10	270	2.51
11	270	2.51
12	270	2.52
13	270	2.52
14	270	2.52
14	266	2.52
14	264	2.53
14	262	2.53
14	260	2.53
15	258	2.53
15	256	2.53
15	254	2.52
15	253	2.52
15	252	2.52
15	251	2.52
15	250	2.52

**Table 2:** Temperature/displacement/time data for SPS of Al—7Zn—3Sn at 300 °C, 40 MPa.

Time (mins)	Temperature (˚C)	Displacement (mm)
0	250	0
1	250	0
2	250	0
3	250	0
4	250	0
5	250	2
6	250	2.3
6.1	251	2.3
6.2	252	2.4
6.3	254	2.3
6.4	256	2.5
6.5	260	2.3
6.6	264	2.5
6.7	265	2.6
6.8	267	2.4
6.9	268	2.5
7	270	2.6
7.1	270	2.6
7.1	271	2.6
7.1	271	2.6
7.2	273	2.6
7.2	274	2.6
7.2	272	2.6
7.2	275	2.6
7.2	277	2.6
7.2	274	2.6
7.3	276	2.6
7.4	278	2.7
7.5	280	2.7
7.5	283	2.7
7.5	281	2.7
7.5	283	2.7
7.5	285	2.7
7.6	286	2.7
7.6	288	2.6
7.7	288	2.7
7.8	289	2.6
8	290	2.7
9	300	2.8
10	301	2.9
11	302	3
12	303	3
13	304	3
14	304	3
14	304	3
14	303	3
14	302	3
14	301	3
15	300	3
15	300	3
15	280	3
15	290	3
15	280	3
15	270	3
15	260	3

**Table 3:** Temperature/displacement/time data for SPS of Al—7Zn—3Sn at 350 °C, 40 MPa.

Time (mins)	Temperature (˚C)	Displacement (mm)
0	250	0
1	250	0
2	250	0
3	250	0
4	250	0
5	250	1.6
6	250	2.3
6.1	255	2.3
6.2	256	2.3
6.3	257	2.3
6.4	258	2.3
6.5	260	2.3
6.6	260	2.4
6.7	260	2.4
6.8	265	2.4
6.9	270	2.4
7	275	2.4
7.1	280	2.4
7.1	286	2.5
7.1	288	2.5
7.2	289	2.5
7.2	290	2.5
7.2	291	2.5
7.2	292	2.5
7.2	294	2.6
7.2	290	2.6
7.3	296	2.6
7.4	295	2.6
7.5	300	2.6
7.5	303	2.6
7.5	310	2.6
7.5	317	2.7
7.5	320	2.7
7.6	324	2.7
7.6	330	2.7
7.7	335	2.7
7.8	340	2.7
8	345	2.7
9	350	2.8
10	350	2.8
11	350	2.9
12	350	3.2
13	350	3.4
14	350	3.4
14	351	3.4
14	352	3.4
14	353	3.4
14	354	3.4
15	350	3.4
15	290	3.4
15	280	3.4
15	270	3.4
15	269	3.4
15	268	3.4
15	260	3.4

**Table 4:** Temperature/displacement/time data for SPS of Al—10Zn—5Sn at 350 °C, 40 MPa.

Time (mins)	Temperature (˚C)	Displacement (mm)
0	250	0
1	250	0
2	250	0
3	250	0
4	250	0
5	250	1.6
6	250	2.3
6.1	255	2.3
6.2	256	2.4
6.3	257	2.5
6.4	258	2.6
6.5	260	2.7
6.6	260	2.7
6.7	260	2.8
6.8	265	2.8
6.9	270	2.9
7	275	3
7.1	280	3
7.1	286	3
7.1	288	3
7.2	289	3
7.2	290	3
7.2	291	3
7.2	292	3.1
7.2	294	3.1
7.2	290	3.1
7.3	296	3.1
7.4	295	3.1
7.5	300	3.1
7.5	303	3.1
7.5	310	3.2
7.5	317	3.2
7.5	320	3.2
7.6	324	3.2
7.6	330	3.2
7.7	335	3.2
7.8	340	3.2
8	345	3.3
9	350	3.4
10	350	3.8
11	350	3.8
12	350	3.8
13	350	3.8
14	350	3.8
14	351	3.8
14	352	3.8
14	353	3.8
14	354	3.8
15	350	3.8
15	290	3.9
15	280	3.9
15	270	3.9
15	269	3.9
15	268	3.9
15	260	3.9

**Table 5:** Temperature/displacement/time data for SPS of Al—13Zn—7Sn at 300 °C, 40 MPa.

Time (mins)	Temperature (˚C)	Displacement (mm)
0	250	0
1	250	0
2	250	0
3	250	0
4	250	0
5	250	1.6
6	250	1.8
6.1	253	2
6.2	255	2.2
6.3	256	2.3
6.4	258	2.4
6.5	260	2.5
6.6	264	2.6
6.7	265	2.8
6.8	266	2.9
6.9	268	3
7	270	3.1
7.1	271	3.1
7.1	272	3.1
7.1	273	3.1
7.2	275	3.2
7.2	276	3.2
7.2	278	3.2
7.2	281	3.2
7.2	283	3.3
7.2	284	3.3
7.3	285	3.3
7.4	287	3.3
7.5	289	3.3
7.5	290	3.4
7.5	291	3.4
7.5	293	3.4
7.5	294	3.4
7.6	295	3.4
7.6	297	3.5
7.7	298	3.5
7.8	299	3.5
8	300	3.5
9	300	3.6
10	300	3.6
11	300	3.7
12	300	3.7
13	300	3.7
14	300	3.8
14	300	3.8
14	300	3.8
14	300	3.8
14	290	3.8
15	280	3.8
15	280	3.8
15	270	3.8
15	270	3.8
15	260	3.8
15	260	3.8
15	260	3.8

**Table 6:** Temperature/displacement/time data for SPS of Al—13Zn—7Sn at 350 °C, 40 MPa.

Time (mins)	Temperature (˚C)	Displacement (mm)
0	250	0
1	250	0
2	250	0
3	250	0
4	250	0
5	250	1.3
6	250	2.6
6.1	255	2.6
6.2	260	2.7
6.3	265	2.7
6.4	268	2.7
6.5	270	2.8
6.6	275	2.8
6.7	280	2.8
6.8	285	2.9
6.9	288	2.9
7	290	3
7.1	291	3
7.1	292	3
7.1	293	3
7.2	294	3.1
7.2	295	3.1
7.2	296	3.1
7.2	297	3.1
7.2	298	3.1
7.2	299	3.1
7.3	300	3.1
7.4	301	3.2
7.5	302	3.2
7.5	303	3.2
7.5	304	3.2
7.5	305	3.2
7.5	306	3.2
7.6	307	3.2
7.6	308	3.3
7.7	308	3.3
7.8	309	3.3
8	310	3.3
9	330	3.6
10	350	3.8
11	350	3.8
12	350	3.8
13	350	3.8
14	350	3.8
15	350	3.8
15	350	3.8
15	350	3.8
15	350	3.8
16	350	3.8
16	305	3.8
16	300	3.8
18	294	3.8
18	283	3.8
18	272	3.8
18	260	3.8

**Table 7:** Density, Relative density and Porosity data of sintered samples.

Samples with sintering (temperature and pressure)	Density (g/cm3)	Relative density (%)	Porosity (%)
Al—7Zn—3Sn (270 °C, 30 MPa)	2.477	86.3	13.7
Al—7Zn—3Sn (300 °C, 40 MPa)	2.678	93.3	6.7
Al—7Zn—3Sn (350 °C, 40 MPa)	2.837	98.9	1.1
Al—10Zn—5Sn (300 °C, 40 MPa)	2.642	89.2	10.8
Al—10Zn—5Sn (350 °C, 40 MPa)	2.689	90.8	9.2
Al—13Zn—7Sn (300 °C, 40 MPa)	2.679	87.8	12.2
Al—13Zn—7Sn (350 °C, 40 MPa)	3.052	99.7	0.3

**Table 8:** Micro hardness and Tensile strength data.

Samples	Micro hardness HV	Micro hardness (MPa)	Tensile strength (MPa)
Al—7Zn—3Sn (300 °C, 40 MPa)	46.53	456.32	149.80
Al—7Zn—3Sn (350 °C, 40 MPa)	55.20	541.35	177.71
Al—10Zn—5Sn (300 °C, 40 MPa)	56.61	555.17	182.25
Al—10Zn—5Sn (350 °C, 40 MPa)	58.42	572.92	188.08
Al—13Zn—7Sn (300 °C, 40 MPa)	50.44	494.67	162.39
Al—13Zn—7Sn (350 °C, 40 MPa)	45.92	450.34	147.84

## 2 Experimental design, materials, and methods

The experiment was designed as follows: Three sets of samples were planned; Al—7Zn—3Sn, Al—10Zn—5Sn, and Al—13Zn—7Sn. Sintering parameter of interest was Temperature (300 and 350 °C) even though a sample was sintered at 270 °C, 30 MPa for comparison. Other parameters that were held constant include (dwell time: 5 mins, pressure: 40 MPa, heating rate: 20 °C/mins). So, a total of six samples were designed to be sintered. They include Al—7Zn—3Sn, Al—10Zn—5Sn and Al—13Zn—7Sn (sintered at 300 °C, 40 MPa, 5 mins and 20 °C/mins) and Al—7Zn—3Sn, Al—10Zn—5Sn and Al—13Zn—7Sn (sintered at 350 °C, 40 MPa, 5 mins and 20 °C/mins).

### 2.1 Materials

Aluminium (Al) of 45 μm particle size, 98.5% purity; Zinc (Zn) 60–100 μm particle size, 98% purity and Tin (Sn) of 60 μm particle size, 98.9% purity powders were used in this work to produce alloys of Al—Zn—Sn. They were supplied by HONGWU international Group, China. The densities and melting points of the metals as reported in the data sheet include Al: 2.7 g/cm3, 660.32 °C; Zn: 7.14 g/cm3, 419.53 °C; Sn: 5.769, 219.93 °C respectively.

### 2.2 Method

Consolidation method was SPS. Raw powders of Al, Zn and Sn were weighed out as required to produce 20 mm diameter Al—Zn—Sn composites. SPS samples are usually small (20–40 mm in diameter) so as to minimize temperature gradient across the sample during sintering. Before sintering commenced, the powders were measured into a plastic container and blended with a tubular mixer (in dry mode at a speed of 110 rpm for 10 hours) to ensure homogeneous mixing [1]. Then the ad-mixed powders were weighed into a die, pressed lightly with a hydraulic press and covered with a graphite cap of which the die is made of. Then the die with its content was placed inside the sintering chamber and locked up. The sintering parameters as specified on the Table 1, Table 2, Table 3, Table 4, Table 5, Table 6 were inputted and the machine switched on (dwell time of 5 mins and a heating rate of 20 °C/mins were used on all the samples). As the machine was switched on, the sintering chamber was evacuated so as to remove possible reactive gasses which could contaminate the material being sintered. After the evacuation, start button was pushed on and the sintering begun. As could be seen in Fig. 1, Fig. 2, Fig. 3, Fig. 4, Fig. 5, Fig. 6, the operation going on inside the chamber could be described as follows: it started with constant heating at the temperature of 250 °C for 4–6 mins which was initialization of the system. Then, there was steady increase of temperature at the rate of 20 °C/mins for the next 4–6 min from the 250 °C to the sintering temperature (300 or 350 °C as the case may be). On reaching the sintering temperature, isothermal heating begun for 5 mins (the dwell time); followed by cooling the sample. After sintering the samples, they were subjected to metallographic preparation where they were cut, grinded and polished to expose the microstructure and remove contamination.
